# Foreign Body in Kidney Presenting as Renal Stone

**DOI:** 10.7759/cureus.11923

**Published:** 2020-12-05

**Authors:** Salman Jamil, Imran K Jalbani, Wajahat Aziz, Syed Raziuddin Biyabani

**Affiliations:** 1 Urology, The Aga Khan University, Karachi, PAK; 2 Surgery, The Aga Khan University, Karachi, PAK

**Keywords:** kidney, foreign body, renal stone

## Abstract

Retained foreign bodies in the urinary system are commonly found in the urinary bladder but are a rare finding in the renal pelvis. Foreign objects can reach the renal pelvis via different pathways. The presence of a foreign body could be iatrogenic, or via direct penetration from the gastrointestinal tract. Presentation could mimic tumor, abscess, hematoma, or even renal stone. We report an unusual case of intrarenal foreign body presenting as renal stone. The patient presented with flank pain and symptoms suggestive of renal stone and a non-contrast CT scan was also indicative of stone, however on endoscopy a foreign body was identified and retrieved.

## Introduction

Foreign bodies in the urinary system are not uncommon. The most common cause is iatrogenic. Forgotten ureteric stents and intrauterine devices are sometimes found in the bladder. Self-insertion of foreign bodies in the urethra has also been described. However, due to protected anatomical location of the kidney, isolated presence of a foreign body in the renal pelvis is a rare occurrence. Kidneys are well protected by the lower two ribs, abdominal wall, and paraspinal muscles [1]. Anteriorly, intraperitoneal contents provide protection. The commonest source of these foreign bodies is iatrogenic, i.e. leftover after intrabdominal or renal surgery. Less common routes reported are migration from GI tract and rare reports suggest retrograde migration of foreign body from the urethra to the kidney [2].

A high index of suspicion is needed especially in atypical presentation of renal stones. Patients may present with recurrent infection, renal colic, and abscess formation or sometimes a foreign body in the kidney may mimic a renal tumor [3,4]. Abdominal sponges and gauze have radiopaque markers hence can easily be seen on plain abdominal radiographs. Often computerized tomography is required (with or without contrast) to reach the diagnosis [5]. We are reporting a case of a renal foreign body presenting as a calculus.

## Case presentation

**Figure 1:**
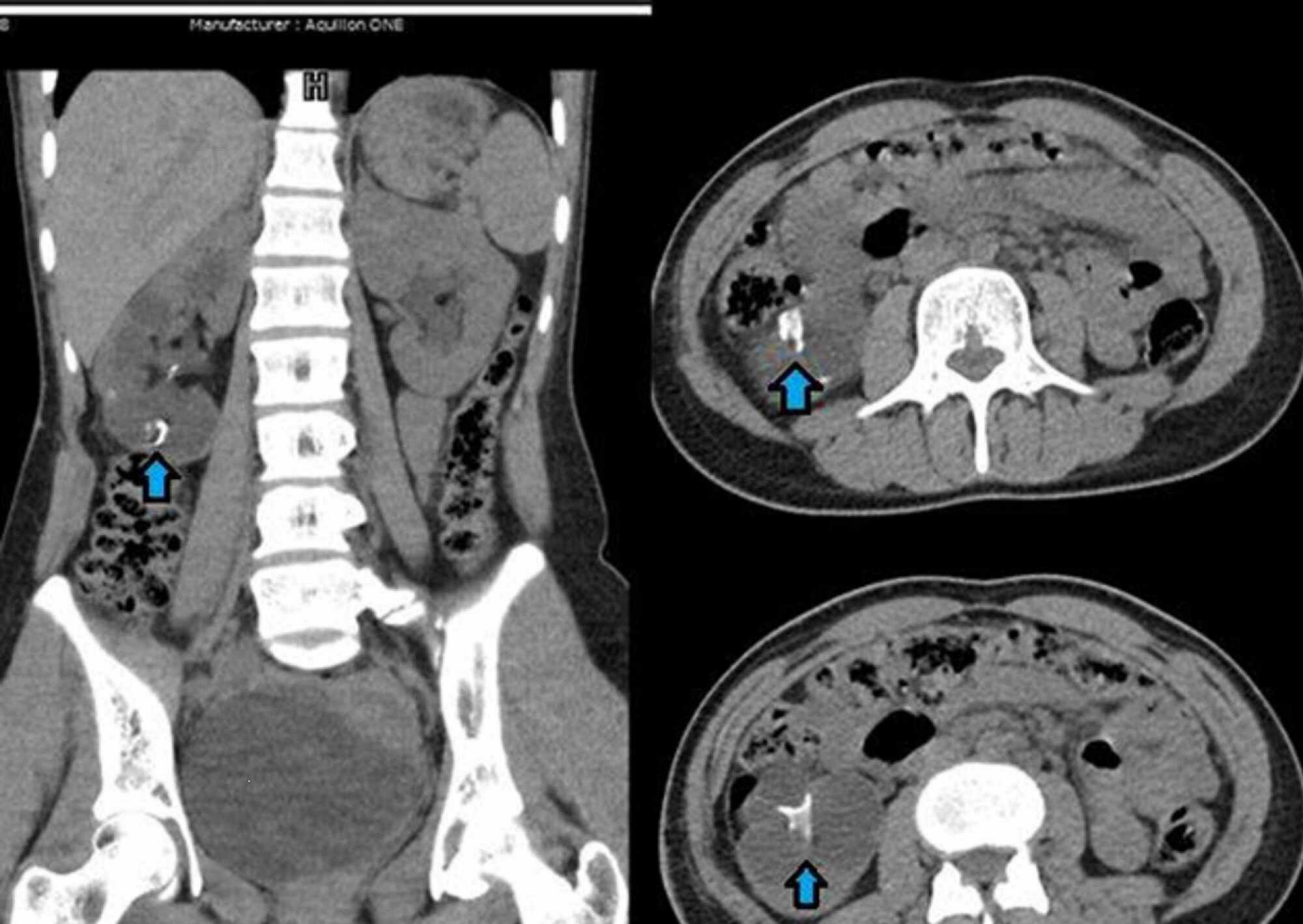
Coronal and Axial images of CT KUB (kidney, ureter, and bladder) X-ray: showing hyperdense circular density in lower pole of right kidney (blue arrow)

An eighteen-year-old woman presented in the outpatient department for intermittent right lumbar pain associated with nausea for two months. She denied any history of fever, hematuria or stone passage. Past history was significant for right open pyelolithotomy four years back. According to patient, no ureteric stent was placed at the time of surgery. Her examination revealed a healed scar in the right flank region, no tenderness or discharging sinus was appreciated. An unenhanced CT abdomen showed a density representing calculus at the lower pole of the right kidney measuring 18 x 9mm (Figure 1). The left kidney and both ureters were unremarkable. 

**Figure 2:**
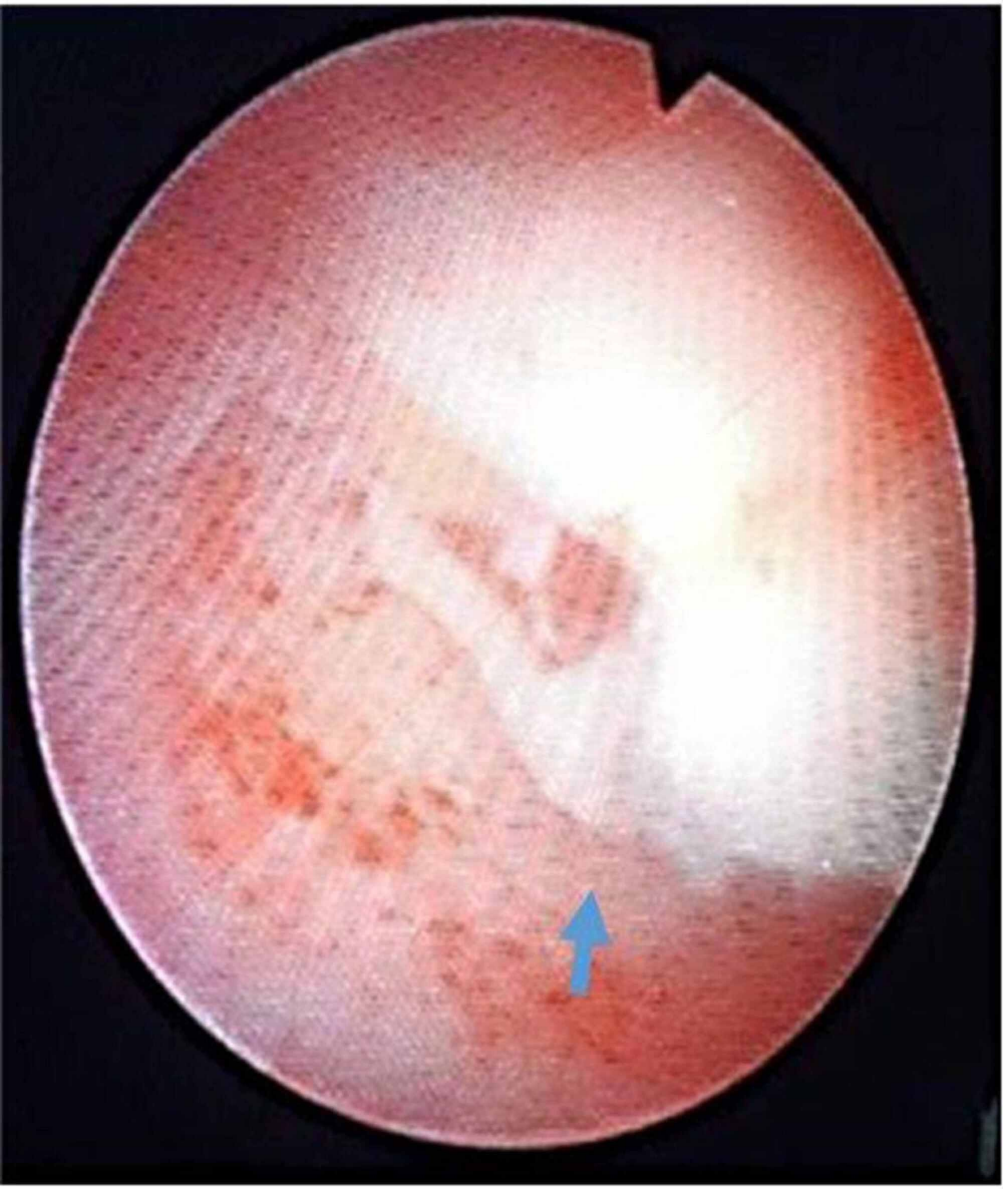
Endoscopic view of foreign body embedded in the mucosa of lower pole calyx (blue arrow)

After thorough discussion with the patient regarding available options, she opted for right percutaneous nephrolithotomy (PCNL). Percutaneous nephrolithotomy was performed in a prone position. Lower pole posterior calyx was punctured and tract dilated up to 21French using metallic dilators and 22Fr Amplatz sheath was placed under fluoroscopic guidance. However surprisingly no stone was appreciated endoscopically after a thorough search in the lower pole calyx and rest of the collecting system (Figure 2).

**Figure 3:**
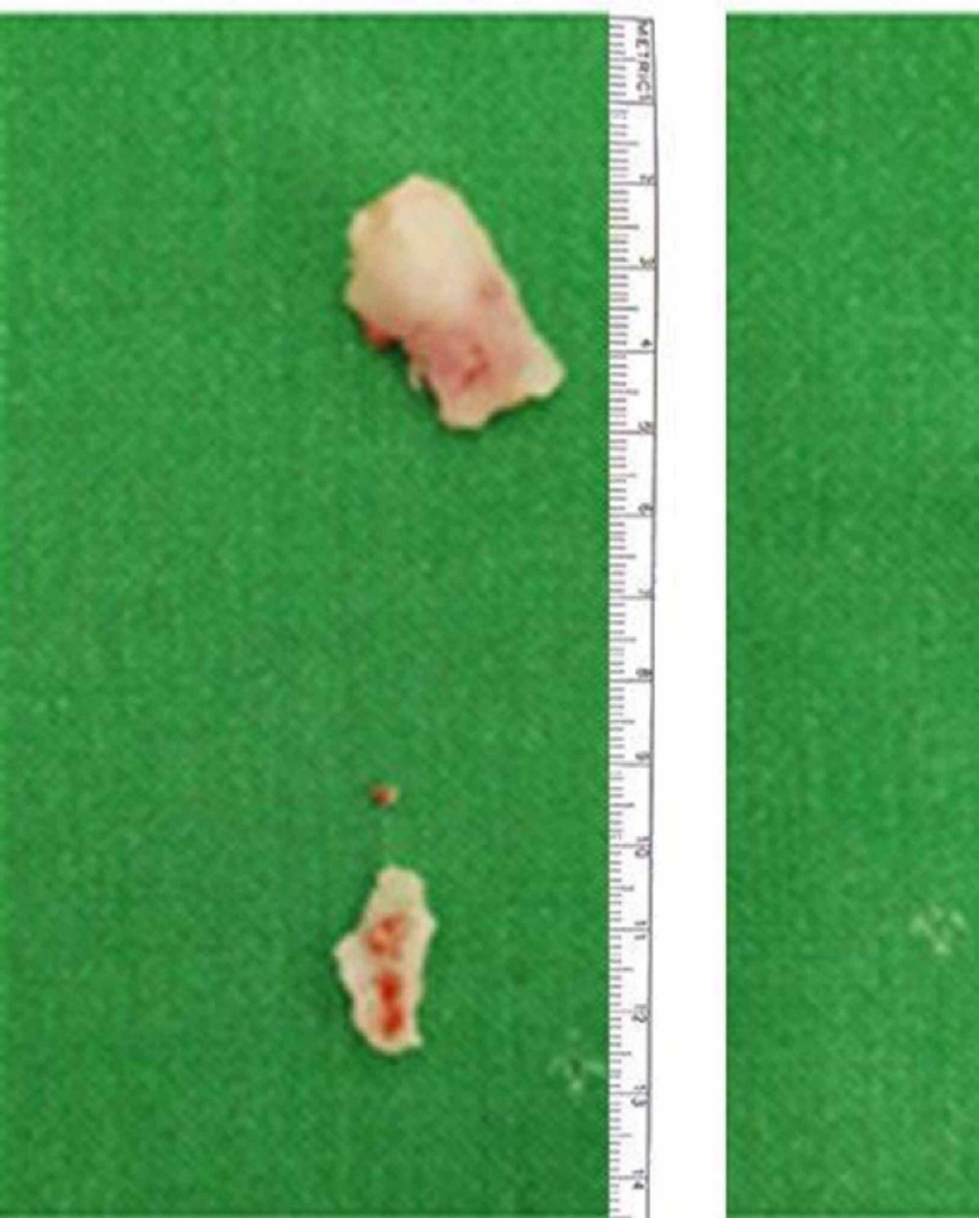
Rubber tube pieces after extraction.

After an extensive search and fluoroscopic guidance, a foreign body was found embedded in the mucosa of the lower pole calyx. This was approximately 1.5cm in size and removed with forceps in pieces with great difficulty. Percutaneous nephrostomy tube (PCN) was placed at the end of the procedure. On careful inspection of the foreign body, it turned out to be a piece of rubber tubing similar to abdominal drains. It was postulated to be a broken drain piece left during previous pyelolithotomy (Figure 3). 

Our patient underwent non-contrast-enhanced computerized tomography (Figure 1). Currently a contrast enhanced imaging is advisable for patients requiring intervention for renal stone [6]. We supplemented non-contrast-enhanced CT with per-operative retrograde pyelography to plan endoscopic access and define intrarenal anatomy. As stone formation in this young patient was most likely secondary to foreign body formation we have not performed 24-hour urine studies. However, she had basic biochemistry including serum creatinine, electrolytes, uric acid, calcium; besides urinalysis and culture which were unremarkable. 

The preliminary diagnosis was renal stone and even on preoperative CT scan foreign body was not identifiable due to a similar density of drain and renal stones. As there was a previous history of open renal surgery, a forgotten double-J stent was a possibility, however, there was no history of ureteric stent insertion during the previous pyelolithotomy nor was a foreign body visible on CT scan. In retrospect on the coronal section of the CT scan, one can make out a rounded outline of a broken drain piece (Figure 1).

The patient had a postoperative smooth recovery and became asymptomatic. She was followed with kidney, ureter, and bladder (KUB) X-ray which confirmed stone and foreign body free status.

## Discussion

The commonest route of an intrarenal foreign body is iatrogenic or traumatic penetration from outside the body, i.e. bullets or shrapnel. In atraumatic cases, migration from the gastrointestinal tract is commonly suggested. The other less common or unexplainable route is retrograde migration from the bladder. Foreign bodies are typically lacerating or pointed objects such as fishbone, needles, pins, hairgrips, toothpicks [7].

Leftover objects after renal surgery are rarely reported in literature and reports of the intrarenal foreign bodies are scarce. Clinical presentation varies from asymptomatic cases to abscess formation, cutaneous fistula, tumor, or renal stone.

In our case, the expectation of having a renal foreign body was very low as the scan was not suggestive of a foreign body. A high index of suspicion is required especially in the evaluation of previously operated patients. 

The typical radiographic appearance on CT or MRI can assist in the diagnosis. In hospitals where radiopaque markers are used with swabs and sponges, a simple plain radiograph can be helpful in making the diagnosis. Surgical gauze has a whorled appearance on the CT or T2 weighted MRI images. However, they cannot reliably distinguish retained surgical sponge from other urological disorders like abscesses, tumors, or stones [8]. Removal of a retained foreign body is mandatory via open surgery or endoscopically due to the high complication rate if left untreated. In the literature, various reports of endoscopic removal of retained intrarenal sponge are reported. These objects were taken out with grasping forceps, dormia basket, or laparoscopic scissor [9].

We were able to manage our patient successfully with a percutaneous approach to the kidney. Plastic tube pieces were removed using grasping forceps. As plastic tube piece was removed with prior history of ipsilateral renal surgery. The plastic material was similar to the one used in abdominal drains so in all likelihood it was a broken piece of a drain that subsequently migrated in the kidney or was inappropriately placed.

Prevention of these occurrences is always better than treating the adverse events resulting from retained foreign bodies and hence strict safeguard measures can ensure such prevention. Improved perioperative patient processing, i.e. sign-in, time in, time out, sign out, swab, needle/suture count and better provider-to-provider communication can minimize these incidences. Sponges should be used one by one. Before closing the incision, the surgeon should explore the whole cavity. All the appliances and surgical sponges should be counted by two or more nurses, the circulating and the scrub nurses. Small sponges should be avoided and only sponges with radiopaque markers should be used. A plain X-ray at the end of operation would be helpful [9].

## Conclusions

Renal foreign bodies are a rare entity. A high index of suspicion is needed to diagnose them as even radiologists can easily miss the diagnosis. 
